# Levothyroxine‐Associated Thyrotoxicosis and Hyperbilirubinemia on Preoperative Screening Evaluation

**DOI:** 10.1155/crie/4776246

**Published:** 2025-12-22

**Authors:** Carly J. Suriano, Ian P. Erkkila, Thomas M. Vara

**Affiliations:** ^1^ Department of General Surgery, OhioHealth Riverside Methodist Hospital, Columbus, Ohio, USA, ohiohealth.com; ^2^ Department of General Surgery, Chalmers P. Wylie Veterans Ambulatory Care Center, Columbus, Ohio, USA

## Abstract

**Background:**

Levothyroxine is a commonly prescribed medication for treatment of hypothyroidism in the United States. This drug is titrated based on weight and serial laboratory evaluation and is often very well tolerated with few adverse effects. Levothyroxine‐associated liver injury and cholestatic disease are regarded as rare phenomena. We report an additional case of levothyroxine‐associated hepatic dysfunction with resolution upon decreased dosage and continued tolerance of the medication.

**Case Presentation:**

We report a case of a 60‐year‐old male presenting for preoperative clearance for umbilical hernia repair with previous history of thyroidectomy for multinodular goiter. Laboratory work‐up suggested thyrotoxicosis‐associated hepatic dysfunction secondary to levothyroxine overtreatment with associated mixed hepatocellular and cholestatic pattern of injury. Extensive work‐up failed to reveal other etiology of liver dysfunction and levothyroxine overtreatment was regarded as the likely cause. Complete resolution of the elevated hepatic enzymes occurred with decreased levothyroxine dosing and return of euthyroid state.

**Conclusion:**

Although regarded as a rare phenomenon, levothyroxine use and drug‐induced thyrotoxicosis should be considered as a possible etiology when evaluating hepatic dysfunction. This case highlights that levothyroxine may be safely continued following a dosing adjustment with noted resolution of hepatic enzyme abnormalities in certain clinical scenarios.

## 1. Introduction

The thyroid hormones, thyroxine and triiodothyronine, are essential for the regulation of hepatocyte metabolic rate and modulation of hepatic function [1, 2]. It is estimated that nearly 5% of Americans aged 12 years or older suffer from hypothyroidism [3]. Potential etiologies include iodine deficiency, autoimmune disorders, congenital conditions, or patients who have previously undergone thyroidectomy or radioactive iodine ablation therapy [3]. Levothyroxine, a commonly prescribed medication for the treatment of hypothyroidism, is typically regarded as a well‐tolerated medication when correct weight‐based dosing is achieved [4]. Levothyroxine‐associated liver injury and cholestatic disease have been previously described but continue to be regarded as a rare adverse effect [5–7]. This report seeks to document a further self‐limited case of such phenomenon with resolution of liver enzyme abnormality following proper dosing adjustment.

## 2. Case Presentation

We report a 60‐year‐old male with a pertinent history of hypertension, hyperlipidemia, gastroesophageal reflux disease, obstructive sleep apnea, metabolic dysfunction‐associated steatotic liver disease (MASLD), posttraumatic stress disorder, and ulcerative proctitis being managed on mesalamine. The patient’s body mass index is 28 kg/m^2^, with no known history of diabetes mellitus. Notable daily medications included high dose atorvastatin as well as social history of 1–2 alcoholic beverages monthly or less. He was previously diagnosed with a multinodular goiter and underwent total thyroidectomy in 2021 with limited central neck dissection and no abnormal lymph nodes identified. Surgical pathology confirmed benign adenomatous hyperplasia and Hashimoto’s thyroiditis. He presented for general surgery evaluation in August 2024 for umbilical hernia repair which prompted presurgical medical clearance. Historically during a previous endocrinology visit in April 2023, the patient’s thyroid‐stimulating hormone (TSH) was noted to be significantly elevated to 52.7 mcIU/mL (normal range 0.27–4.20 mcIU/mL), with free T4 at the low end of normal (0.77 ng/dL, normal range 0.7–1.7 ng/dL). His levothyroxine dosing was increased to 175 mcg daily (1.6 mcg/kg) at that time. Following this, the patient was lost to follow‐up until he re‐established care with his endocrinologist in August 2024 at the time of his presurgical evaluation. The patient did report heat intolerance as well as mild fine tremors. He denied any jaundice or other acute symptoms. Interestingly, the patient reported an intentional 20–30‐pound weight loss over the previous months but had not had an adjustment made in levothyroxine dosage. Notably, his TSH during his preoperative clearance visit was suppressed to 0.02 mcIU/mL with elevated free T4 to 2.15 ng/dL. Additionally, the patient was found to have elevated transaminases with aspartate aminotransferase (AST) of 98 U/L and alanine aminotransferase (ALT) 182 U/L as well as elevated alkaline phosphatase to 138 U/L and hyperbilirubinemia with total bilirubin of 1.50 mg/dL and direct bilirubin of 0.7 mg/dL. Thyroid and hepatic function laboratory results throughout the preoperative period are displayed in Table 1. The patient was seen and evaluated by endocrinology and gastroenterology. Abdominal MRI was obtained and was negative for choledocholithiasis, fatty liver changes, or evidence of biliary duct pathology. Right upper quadrant ultrasound was also negative for evidence of biliary pathology. Acute hepatitis panel was negative without any known prior history of hepatitis A or B exposure. Additionally, serum iron studies, anti‐smooth muscle antibody, ceruloplasmin, and antimitochondrial antibody all resulted within normal limits, aside from an elevated ferritin to 1226 ng/mL. Results of further extensive dedicated laboratory work‐up are shown in Table 2. His levothyroxine dose was decreased to 150 mcg daily (1.58 mcg/kg) and again decreased to 125 mcg daily (1.42 mcg/kg) at a subsequent visit. Interval labs demonstrated resolution of the previously noted liver function abnormalities as well as correction of TSH and free T4 to normal limits, 0.61 mcIU/mL and 1.30 ng/dL, respectively. The patient ultimately proceeded with an umbilical hernia repair with an uneventful postoperative course.

**Table 1:** Patient’s thyroid and hepatic function laboratory results are displayed.

Laboratory test	May 3, 2022	April 13, 2023	June 24, 2024	August 14, 2024	September 3, 2024	September 17, 2024	October 22, 2024	December 12, 2024
TSH (mIU/L)	0.06	52.70	—	14.65	0.13	0.06	0.02	0.61
Free T4 (ng/dL)	—	0.77	—	1.43	1.91	1.83	2.15	1.30
T3 (ng/dL)	—	—	—	—	—	—	—	—
T‐Bili (mg/dL)	0.60	0.70	0.80	—	1.50	1.30	1.20	0.7
D‐Bili (mg/dL)	0.2	0.3	0.2	—	0.7	0.6	0.4	0.2
Alk Phos (IU/L)	88	106	92	—	138	149	162	110
ALT (U/L)	57	87	104	—	182	187	87	38
AST (U/L)	45	61	71	—	98	92	48	27
GGT (IU/L)	—	—	—	—	—	46	—	—
Serum albumin (g/dL)	4.0	4.5	4.1	—	4.3	4.2	4.0	3.9

Abbreviations: Alk Phos, alkaline phosphatase; ALT, alanine aminotransferase; AST, aspartate aminotransferase; D‐bili, direct bilirubin; GGT, gamma‐glutamyl transferase; T‐bili, total bilirubin; TSH, thyroid‐stimulating hormone.

**Table 2:** Results of further dedicated laboratory work‐up intended to identify etiology of hepatic dysfunction.

Laboratory test	September 17, 2024	October 22, 2024
Iron (mcg/dL)	91	—
TIBC (mcg/dL)	316	—
Iron % Sat (%)	29	—
Ferritin (μg/L)	1226	—
Ceruloplasmin (mg/dL)	33	36
Hep B S‐Ab	Reactive	—
Hep B S‐Ag	Nonreactive	Nonreactive
Hep C Ab	Nonreactive	Nonreactive
Hep A Ab IgM	Nonreactive	Nonreactive
Hep A Ab	Reactive	—
Hep B core Ab	Nonreactive	—
tTG IgA (U/mL)	<2	—
tTG IgG (U/mL)	<2	—
Anti‐smooth muscle Ab (U)	6	5
ANA IFA	Negative	—
Anti‐mitochondrial Ab	Negative	—
IgA, total (mg/dL)	330	—
Mitochondrial Ab (U/mL)	<20.0	—
Alpha‐1‐antitrypsin (mg/dL)	177	—

*Note:* Ab, antibody; Ag, antigen; Hep A, hepatitis A; Hep B, hepatitis B; Hep C, hepatitis C; Iron % Sat, iron percent saturation; S‐Ab, surface antibody; S‐Ag, surface antigen.

Abbreviations: ANA IFA, antinuclear antibody indirect immunofluorescence assay; TIBC, total iron binding capacity; tTG IgA, tissue transglutaminase IgA; tTG IgG, tissue transglutaminase IgG.

## 3. Discussion

Previous literature has demonstrated an association between thyroid and hepatic function where dysfunction in one system impacts the other [1, 2]. There have been documented cases of thyroid abnormalities in such liver diseases as cirrhosis, chronic hepatitis C, cholangiocarcinoma, or hepatocellular carcinoma [2]. Hepatic dysfunction in hyperthyroidism specifically was first described in 1874 [8]. Previous case reports have documented hyperbilirubinemia with cases of thyrotoxicosis and thyroid storm [6] as well as secondary to the commonly used pharmacologic agents such as methimazole [9, 10] and propylthiouracil [11–13]. Today, it is well documented that up to 60% of patients with Graves’ disease exhibit at least one abnormal LFT, with improvement typically observed following treatment [14]. However, jaundice or hyperbilirubinemia is much less common in *hyper*thyroidism, or conversely, are associated with *hypo*thyroidism‐induced cholestasis [5].

Levothyroxine (Synthroid), a synthetic form of L‐thyroxine (T4), is a commonly used treatment choice for hypothyroidism and is typically very well tolerated [15]. Levothyroxine is one of the most commonly prescribed medications in the United States with more than 100 million prescriptions annually [4]. Side effects are rarely reported if correct dosage is achieved; however, multiple adverse effects are reported if dosage exceeds patients’ metabolic needs, such as weight loss, anxiety, tremor, tachycardia, arrhythmias, and emotional liability to name a few. Elevation in serum hepatic enzyme levels is a less commonly reported association with high doses of levothyroxine [4]. Previous studies have shown that these abnormalities will typically resolve after a euthyroid state is achieved [4]. Monitoring of liver enzymes while on levothyroxine is not routinely recommended as levothyroxine‐associated liver injury is regarded as a rare adverse effect [15].

This case seeks to highlight suspected hyperbilirubinemia and hepatic enzyme derangement in the setting of incorrect levothyroxine dosing. This patient had been treated with levothyroxine since his previous thyroidectomy in 2021, and the drug had been well tolerated until he was lost to follow‐up and underwent an intentional 20–30‐pound weight loss. The patient does have a known history of MASLD and takes a daily statin, both of which could contribute to periodic LFT abnormality. However, recent imaging did not suggest fatty liver disease and notably decreased dosing of his levothyroxine lead to an improvement in the abnormal hepatic enzymes reported. The patient did not demonstrate any evidence of high‐output heart failure secondary to his thyrotoxicosis, therefore, further ruling out passive hepatic congestion. Additionally, although the patient was not evaluated for a possible thyroid remnant which can occur after surgery for multinodular goiter, possible autonomous function within a remnant thyroid or possible remnant thyroiditis was felt to be clinically unlikely as the decline in free T4 was proportional to the reduction in levothyroxine dosage. The patient underwent extensive further evaluation, and no other identifiable etiology of his hepatic enzyme elevation was identified. Other underlying liver diseases were excluded. Previous cases reporting levothyroxine‐associated hepatic dysfunction [7, 15] have demonstrated mainly hepatocellular injury pattern which is regarded as the more common hepatic manifestation of thyroid disease [16]. Interestingly, our patient displayed a mixed hepatocellular and cholestatic pattern. Our patient did have a greater ALT than AST which suggests liver cellular injury and is often seen in certain conditions such as MASLD, medication‐induced liver injury, or viral hepatitis [17]. Whereas, an AST‐predominant pattern is more commonly associated with alcohol use or cirrhosis. A cholestatic pattern typically represents a hepatobiliary cause such as bile duct obstruction, although non‐hepatic causes are also possible such as with pregnancy or congestive heart failure [18]. A mixed hepatocellular and cholestatic pattern as seen in this report has been implicated in drug‐induced liver injury as well as acute viral or autoimmune hepatitis which were subsequently ruled out for this patient [19]. A Roussel Uclaf Causality Assessment Method (RUCAM) score was calculated with a total of 7 points, suggesting probable medication‐induced liver injury.

The etiology of our patient’s mixed hepatocellular and cholestatic injury pattern is likely multifactorial. In hyperthyroidism (or thyrotoxicosis), excess thyroid hormone directly increases hepatic metabolic activity, which can result in oxidative stress and hepatocyte injury. This in turn can indirectly cause hepatocyte dysfunction and subsequent cholestasis, resulting in a cholestatic injury pattern [2]. Some studies indicate that excessive thyroid hormone may directly cause dysregulation in biliary cholesterol and phosphatidylcholine excretion, as well as bile‐salt independent canalicular secretion [20–22]. Nevertheless, hepatocellular injury remains more common than cholestatic injury across studies to date, which we suspect is a reflection of the direct versus indirect pathophysiologic mechanisms, respectively [14, 23, 24].

Previous reports of levothyroxine‐associated liver injury required stopping the medication altogether in favor of changing to triiodothyronine or changing the formulation of the thyroxine, such as swapping from a tablet to a powder [15, 25]. Our patient was able to tolerate continued levothyroxine use once proper dosage was achieved. This suggests against acquired antigenicity as is noted in previous case reports [7, 26]. Given this reason, it is suspected that the bilirubin increase was more likely due to iatrogenic thyrotoxicosis rather than levothyroxine use itself as this medication was previously tolerated well for years and the hyperbilirubinemia resolved once a euthyroid state was again achieved. This case report highlights the importance of monitoring levothyroxine dosage closely in patients with large weight fluctuations. Additionally, we suggest potential utility in checking thyroid function labs if idiopathic hyperbilirubinemia is identified. Clinicians may also consider checking hepatic function labs if a patient is found to have thyrotoxicosis secondary to levothyroxine overtreatment.

## 4. Conclusion

This case report serves as a reminder that drug‐induced thyrotoxicosis should be considered as a possible etiology when evaluating hepatic dysfunction. Additionally, we demonstrate that incorrect dosing of levothyroxine may lend itself to hepatic dysfunction with a mixed hepatocellular and cholestatic pattern of injury. However, this case highlights that levothyroxine was safely continued following a dosing adjustment with noted resolution of hepatic enzyme abnormalities and continued tolerance of the medication.

## Ethics Statement

Written informed consent was obtained from the patient for the publication of this case report.

## Disclosure

The authors have nothing to report.

## Conflicts of Interest

The authors declare no conflicts of interest.

## Funding

No funding was received for this manuscript.

## Data Availability

The data that support the findings of this study are available from the corresponding author upon reasonable request.
